# Disturbed shear stress reduces Klf2 expression in arterial-venous fistulae in vivo

**DOI:** 10.14814/phy2.12348

**Published:** 2015-03-16

**Authors:** Kota Yamamoto, Clinton D Protack, Go Kuwahara, Masayuki Tsuneki, Takuya Hashimoto, Michael R Hall, Roland Assi, Kirstyn E Brownson, Trenton R Foster, Hualong Bai, Mo Wang, Joseph A Madri, Alan Dardik

**Affiliations:** 1 Veterans Affairs Connecticut Healthcare SystemsWest Haven, Connecticut; 2 Vascular Biology & Therapeutics Program, Yale University School of MedicineNew Haven, Connecticut; 3 Department of Surgery, Yale University School of MedicineNew Haven, Connecticut; 4 Division of Vascular Surgery, Department of Surgery, Graduate School of Medicine, The University of TokyoTokyo, Japan; 5 Department of Pathology, Yale University School of MedicineNew Haven, Connecticut; 6 Division of Cancer Biology, National Cancer Center Research InstituteTokyo, Japan

**Keywords:** Arteriovenous fistula, disturbed flow, kruppel-like factor 2, laminar flow, shear stress

## Abstract

Laminar shear stress (SS) induces an antiproliferative and anti-inflammatory endothelial phenotype and increases Klf2 expression. We altered the diameter of an arteriovenous fistula (AVF) in the mouse model to determine whether increased fistula diameter produces disturbed SS in vivo and if acutely increased disturbed SS results in decreased Klf2 expression. The mouse aortocaval fistula model was performed with 22, 25, or 28 gauge needles to puncture the aorta and the inferior vena cava. Duplex ultrasound was used to examine the AVF and its arterial inflow and venous outflow, and SS was calculated. Arterial samples were examined with western blot, immunohistochemistry, and immunofluorescence analysis for proteins and qPCR for RNA. Mice with larger diameter fistulae had diminished survival but increased AVF patency. Increased SS magnitudes and range of frequencies were directly proportional to the needle diameter in the arterial limb proximal to the fistula but not in the venous limb distal to the fistula, with 22-gauge needles producing the most disturbed SS in vivo. Klf2 mRNA and protein expression was diminished in the artery proximal to the fistula in proportion to increasing SS. Increased fistula diameter produces increased SS magnitude and frequency, consistent with disturbed SS in vivo. Disturbed SS is associated with decreased mRNA and protein expression of Klf2. Disturbed SS and reduced Klf2 expression near the fistula are potential therapeutic targets to improve AVF maturation.

## Introduction

Arterial-venous fistulae (AVF) are the preferred conduit for hemodialysis access, yet they have the worst patency of any procedure performed by vascular surgeons. The factors that regulate AVF failure are not well described, and the role of hemodynamic forces in AVF maturation and failure are complex and not well understood (He et al. 2013; Fitts et al. 2014; Lu et al. 2014). The mechanical forces of the flowing blood, such as wall shear stress (SS) and stretch, provide an environment for the vessel that is not static (Lehoux et al. 2006; Anwar et al. 2012; Kwak et al. 2014). Mechanical stretch is produced by intraluminal pressure and affects both endothelial and smooth muscle cells leading to smooth muscle cell and medial hypertrophy and hyperplasia, with distinct changes in contractile and matrix proteins (Levy et al. 1988; Intengan and Schiffrin 2001; Helmke and Davies 2002; McCue et al. 2004). SS is tangential to the endothelium with a magnitude of approximately 1 Pa, much less than the tensile stress in the wall that is generally 300–500 kPa (Kwak et al. 2014). Arterial laminar SS induces an antiproliferative and anti-inflammatory endothelial phenotype by increasing the expression of atheroprotective genes such as Kruppel-like factor 2 (Klf2) and endothelial nitric oxide synthase (eNOS) (Gimbrone 1999; Topper and Gimbrone 1999; SenBanerjee et al. 2004; Chien 2007; Nayak et al. 2011; Bjorck et al. 2012). Disturbed flow, however, induces a dysfunctional endothelial phenotype by inducing pro-inflammatory mediators such as nuclear factor-kappa B (NF-kB) as well as by increasing the expression of genes related to oxidation and proliferation (Chiu and Chien 2011; Davies et al. 2013).

Klf2 is a zinc finger transcription factor, the expression of which is increased by laminar SS in endothelium (Dekker et al. 2002). Klf2 inhibits inflammation by inactivating p65 and downregulating cell adhesion molecules, inhibiting thrombosis and stimulating vasodilation by upregulating eNOS, and inhibiting angiogenesis by repressing VEGFR2 expression (Arkenbout et al. 2003; Fledderus et al. 2007; Zhang and Friedman 2012; Novodvorsky and Chico 2014). In vitro studies suggest that disturbed SS downregulates Klf2 expression (Wang et al. 2006, 2012; Ni et al. 2010; Lee et al. 2012). Although there are several models of disturbed SS in vivo, (Li et al. 2007; Nam et al. 2009; Shin et al. 2013) the effects of acutely disturbed SS on Klf2 expression in vivo are not well described (Nigro et al. 2011). We recently described a mouse AVF model that mimics human AVF maturation (Yamamoto et al. 2013a,b). This model acutely alters SS in vivo and we used it to compare the effects of increasing magnitudes of SS on Klf2 expression in vivo, such as occurs after surgical creation of an AVF for dialysis access. We hypothesized that increased fistula diameter would increase AVF SS magnitude and frequency, resulting in disturbed SS as well as decreased Klf2 expression in vivo.

## Materials and Methods

### Antibodies

Antibodies against mouse CD31 (PECAM-1) used in immunohistochemistry were raised in rabbits and purified as described elsewhere (Pinter et al. 1997). Rabbit polyclonal antibodies against mouse *α*-smooth muscle actin (*α*-actin) (ab5694) and Klf2 (for western blotting, ab189541) were purchased from Abcam (Cambridge, MA). Anti-Klf2 antibodies for immunohistochemistry and immunofluorescence (sc-18690 and LS-B5627) were purchased from Santa Cruz Biotechnology (Dallas, TX) and LifeSpan Biosciences, Inc (Seattle, WA).

### Anesthesia and surgery

All animal studies were performed in strict compliance with Federal guidelines and our Institutional Animal Care and Use Committee approved the protocol. C57BL/6 mice, aged 10 weeks and 20–30 g body weight, were used and appropriate anesthesia and analgesia was given as described previously (Yamamoto et al. 2013a).

Briefly, mice were anesthetized with 2–3% isoflurane in 0.8 L/min oxygen delivered via an isoflurane vaporizer. The aorta and inferior vena cava (IVC) were exposed after a midline laparotomy under general anesthesia. The proximal infrarenal aorta and the distal aorta were dissected for clamp placement and needle puncture, respectively. After clamping the aorta just below the left renal artery, a needle was used to puncture the aorta and through the back wall, into the front wall of the IVC. The needle was immediately withdrawn and the surrounding connective tissue was used for hemostatic compression. Visualization of pulsatile arterial blood flow in the IVC was assessed as a technically successful AVF.

We previously described using a 25-gauge needle to create the AVF (Yamamoto et al. 2013a,b). We also used 22- and 28-gauge needles to produce AVF with twice or half the fistula area, respectively (Table1). Needles larger than 22G were not used since their diameter exceeds the average diameter of the mouse aorta.

**Table 1:** Cross-sectional area according to the needle size

Needle size (gauge)	Diameter (mm)	Cross-sectional area (mm^2^)	Cross-sectional area ratio (to 25G)
21	0.819	2.106	2.53
22	0.718	1.619	1.94
23	0.642	1.294	1.55
24	0.566	1.006	1.21
25	0.515	0.833	1.00
26	0.464	0.676	0.81
27	0.413	0.536	0.64
28	0.362	0.411	0.49
29	0.337	0.357	0.43

All the values are outer lumen size. Cross-sectional area ratio is calculated in reference to the 25G needle. Underlined values are those used in this report.

### Ultrasound

**Figure 1:**
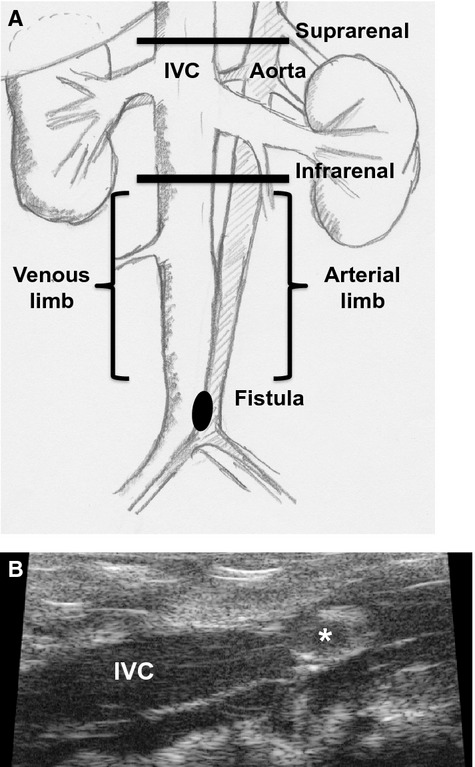
General schema of AVF assessment. (A) The sites of measurement for ultrasound parameters. (B) Ultrasound image showing a longitudinal view of the IVC. High echoic ring (*) represents the fistula facing toward the IVC lumen.

Doppler ultrasound (Vevo770 High Resolution Imaging System; VisualSonics Inc., Toronto, Ontario, Canada) was used to confirm the presence of the AVF and to measure the diameter of and the velocity in the aorta and IVC preoperatively and on days 1, 3, and 7 after surgery (Yamamoto et al. 2013a,b). Waveforms in both the aorta and the IVC were recorded using pulse wave mode and diameters of the aorta and the IVC were determined both above and below the renal arteries (Fig.1A); AVF patency was directly visualized using the pulse wave mode in longitudinal view (Fig.1B). SS was measured by ultrasound and calculated using the Hagen–Poiseuille equation (Kraiss et al. 1991). This equation was used as: SS = 4*η*V/r, where SS is the shear stress, *η* is the blood viscosity, V is the flow velocity in cm/sec, and r is the radius in cm; blood viscosity was assumed to be constant at 0.035 poise.

### Histology

After euthanasia, the circulatory system was flushed under pressure with PBS followed by 10% formalin and the AVF was extracted en bloc. The tissue block was then embedded in paraffin and cut in 5-*μ*m cross sections. Hematoxylin and eosin (H&E) and elastin van Gieson (EVG) staining were performed for all samples. For intima-media thickness measurements, the thickness was measured at eight equidistant points per cross section and averaged.

### Immunohistochemistry

Sections were heated in citric acid buffer (pH 6.0) at 100°C for 10 min for antigen retrieval. The sections were treated with 0.3% hydrogen peroxide in methanol for 30 min at room temperature to block endogenous peroxidase activity and incubated with 5% normal goat serum in PBS (pH 7.4) containing 0.05% Triton X-100 (T-PBS) for 1 h at room temperature to block nonspecific protein-binding sites. Sections were then incubated at 4°C with the primary antibodies diluted at 1:100 (anti-*α*-actin), 1:200 (anti-CD31), and 1:100 (anti-Klf2) in T-PBS. After overnight incubation, the sections were incubated with Dako EnVision™ + Dual Link System-HRP (Dako, Carpinteria, CA) or secondary anti-goat antibody (sc-2020; Santa Cruz Biotechnology, Dallas, TX) for 1 h at room temperature and treated with Dako Liquid DAB+ Substrate Chromogen System (Dako) to visualize the reaction products. Finally, the sections were counterstained with Dako Mayer's Hematoxylin (Lillie's Modification) Histological Staining Reagent (Dako).

### Immunofluorescence

Antigen retrieval was done in the same manner as immunohistochemistry described above. After pretreatment, sections were incubated with 5% normal goat serum in T-PBS for 1 h at room temperature and then incubated at 4°C with primary antibody (anti-Klf2) diluted at 1:100 in T-PBS. After overnight incubation, sections were incubated with secondary antibodies (Alexa Fluor 488-conjugated goat anti-rabbit IgG (H+L), Life Technologies, Grand Island, NY) diluted at 1:100 in T-PBS for 2 h at room temperature. Finally, tissues were counterstained with DAPI (4′,6-diamidino-2-phenylindole) (catalog no. D9564; Sigma-Aldrich, St. Louis, MO) diluted at 1:5000 in T-PBS for 10 min at room temperature and coverslipped with VECTASHIELD Mounting Medium (catalog no. H-1000; Vector Laboratories, Inc., Burlingame, CA). Digital fluorescence images were captured on an Olympus IX71 inverted microscope equipped with a MicroFire Camera and PictureFrame 1.0 software for Macintosh (Optronics, Goleta, CA) with Photoshop CS2 software (Adobe, San Jose, CA) on a Windows 7 computer.

### RNA extraction and quantitative PCR

Total RNA from the arterial limb of the AVF was isolated using the RNeasy Mini kit with digested DNase I (Qiagen); care was taken to avoid surrounding arterial tissue. RNA quality was confirmed by the 260/280 nm ratio. Reverse transcription was performed using the SuperScript III First-Strand Synthesis Supermix (Invitrogen, Carlsbad, CA). Real-time quantitative PCR was performed using SYBR Green Supermix (Bio-Rad Laboratories, Hercules, CA) and amplified for 40 cycles using the iQ5 Real-Time PCR Detection System (Bio-Rad Laboratories). Correct target amplification and exclusion of nonspecific amplification was confirmed by 1.5% agarose gel electrophoresis, and primer efficiencies were determined by melt curve analysis. All samples were normalized by GAPDH RNA amplification.

### Statistical analysis

All data were analyzed using Prism 6 software (GraphPad Software, Inc., La Jolla, CA). Comparison between groups was performed with MANOVA with post hoc tests examined using Dunnett's multiple comparison test. *P* values < 0.05 were considered significant.

## Results

**Figure 2:**
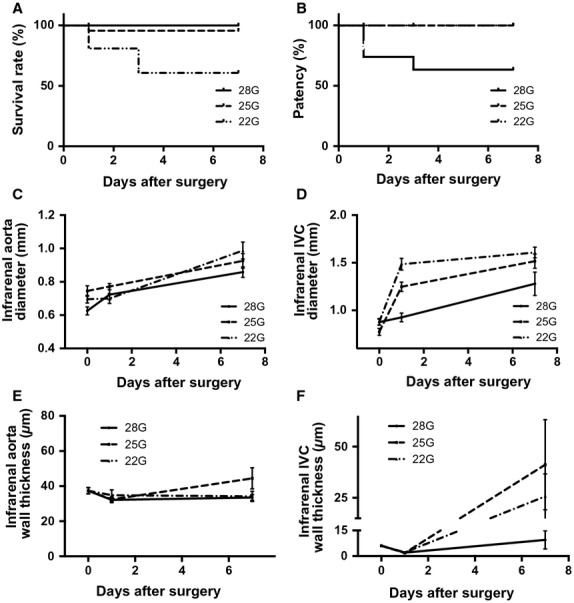
Effects of different diameter fistulae. (A) Cumulative survival after AVF surgery. *n* = 23 for each group; *P* = 0.0024, log rank. (B) Cumulative patency after AVF surgery. *n* = 23 for each group; *P* = 0.0009, log rank. (C) Diameter of the infrarenal aorta after AVF surgery. *n* = 23 for each group; *P* = 0.02 for groups, *P* < 0.0001 for time, Two way ANOVA. (D) Diameter of the infrarenal IVC after AVF surgery. *n* = 23 for each group; *P* < 0.0001 for both groups and time. Two-way ANOVA. (E) Wall thickness of the infrarenal aorta after AVF surgery. *n* = 3 for each group; *P* = 0.27 for groups, *P* = 0.1130 for time, Two-way ANOVA. (F) Wall thickness of the infrarenal IVC after AVF surgery. *n* = 3 for each group; *P* = 0.33 for groups, *P* = 0.0055 for time, Two way ANOVA.

To determine the effect of disturbed SS on Klf2 expression in vivo, we used different diameter needles to make AVF with different fistulae diameters and determined whether larger diameter fistulae resulted in increased magnitudes and frequencies of SS, yielding disturbed SS in vivo. Mice were examined postoperatively with ultrasound to determine fistula patency and compare the survival and patency of mice with fistulae of different diameters; we focused on the first seven postoperative days since we have previously shown that AVF patency is essentially unchanged between postoperative days 7 and 28 in this mouse model, mimicking the maturation phase of human AVF adaptation (Yamamoto et al. 2013b). Although mice with a large diameter fistula (22 gauge) had reduced postoperative survival due to bleeding (day 1) or acute cardiac failure (days 2–3; Fig.2A), they showed increased patency (Fig.2B); conversely, mice with a smaller diameter fistula (28 gauge) had normal postoperative survival but reduced AVF patency.

**Figure 3:**
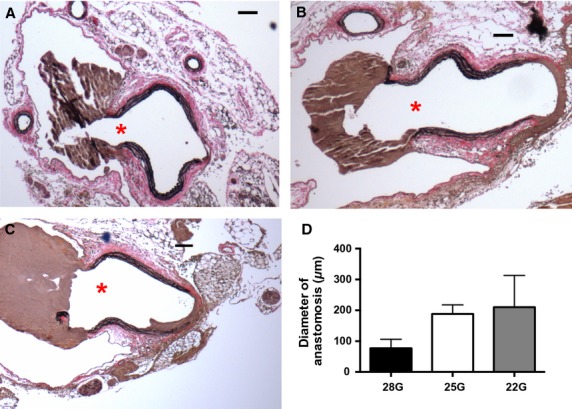
Effects of needle diameter on fistula diameter. (A–C) Representative photomicrographs showing histology of fistulae created using different needle diameters (A, 28G; B, 25G; C, 22G). The red star shows the maximal diameter of the fistula. A small amount of thrombus is seen at the fistula as little flushing was performed in these specimens to prevent anastomotic distention. (D) Mean anastomotic diameter. *n* = 3 for each group; *P* = 0.33.

The diameters of both the infrarenal aorta and the IVC were serially measured using Doppler ultrasound (Fig.2C and D). The diameter of the aorta showed minimal change on the first day but increased gradually over the next 7 days without any significant difference between the AVF of different diameters (Fig.2C). On the other hand, the IVC acutely dilated on the first postoperative day and then increased diameter gradually between postoperative days 2 and 7; unlike the aorta, these diameter increases were proportional to the fistula diameter (Fig.2D). Examining the AVF using histology showed that the AVF wall thickness did not change in the aorta over time (Fig.2E); however, the IVC wall thickness decreased acutely on postoperative day 1 (Fig.2F), consistent with an acute increase of flow and diameter (Fig.2D), and then increased between days 2 and 7 in the larger (22G and 25G) diameter AVF but not in small diameter (28G) AVF (Fig.2F), consistent with AVF maturation (Yamamoto et al. 2013b). To determine if larger diameter needles correlate with larger diameter fistulae in vivo, the diameter of the fistula was examined with histology on postoperative day 1; there was a trend for larger diameter fistulae with larger diameter needle size (Fig.3). These results suggest that larger diameter needles create larger diameter fistulae in vivo, and the resultant hemodynamic changes are established within 1 day of surgery.

**Figure 4:**
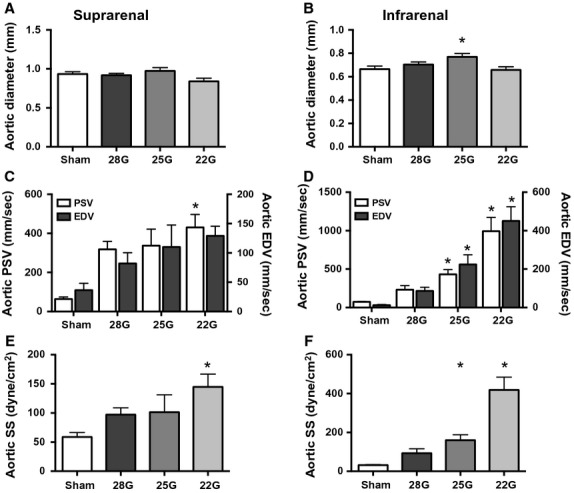
Ultrasound measurements in the aorta proximal to the fistula. Ultrasound derived measurements in the suprarenal (left column) and infrarenal (right column) aorta; diameter (A,B), velocity (C,D), and calculated SS (E,F). In all graphs, *n* = 7 for shams and 5 for the fistulae groups. ANOVA results: A, *P* = 0.09; B, *P* = 0.03; C, *P* = 0.035 (PSV) and *P* = 0.25 (EDV); D, *P* < 0.0001 (PSV) and *P* < 0.0001 (EDV); E, *P* = 0.0235; F, *P* < 0.0001. For all figures, *denotes *P* < 0.05 compared to sham group, post hoc test. PSV, peak systolic velocity; EDV, end-diastolic velocity.

**Figure 5:**
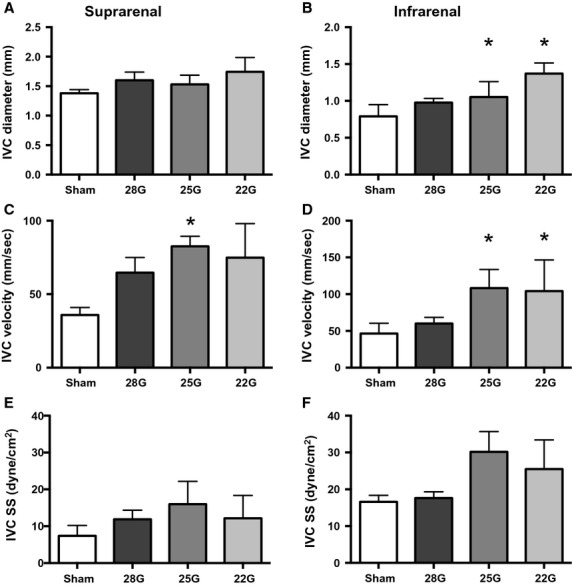
Ultrasound measurements in the IVC distal to the fistula. Ultrasound derived measurements in the suprarenal (left column) and infrarenal (right column) IVC; diameter (A,B), velocity (C,D), and calculated SS (E,F). In all graphs, *n* = 7 for shams and 5 for the fistulae groups. ANOVA results: A, *P* = 0.38; B, *P* = 0.0001; C, *P* = 0.05; D, *P* = 0.0012; E, *P* = 0.07; F, *P* = 0.15. For all figures, *denotes *P* < 0.05 compared to sham group, post hoc test.

Ultrasound was used to measure the SS in the aorta proximal to the fistula and in the IVC distal to the fistula. In the aorta proximal to the fistula, the suprarenal and infrarenal aortic diameters did not change significantly on the first postoperative day (Fig.4A and B), consistent with little acute change in arterial compliance after sudden increase in blood flow. However, peak systolic velocity (PSV) increased proportionally to fistula diameter (Fig.4C and D), consistent with decreased resistance distal to the fistula; the PSV in the infrarenal aorta was greater than the PSV in the suprarenal aorta, consistent with closer proximity to the fistula. The end-diastolic velocity (EDV) similarly increased (Fig.4C and D). Calculated SS magnitude increased proportionally to fistula diameter, both in the suprarenal (Fig.4E) and the infrarenal aorta (Fig.4F). Interestingly, in the infrarenal IVC, both the diameter and the PSV increased on postoperative day 1, resulting in no significant change of SS at any fistula diameter (Fig.5).

**Figure 6:**
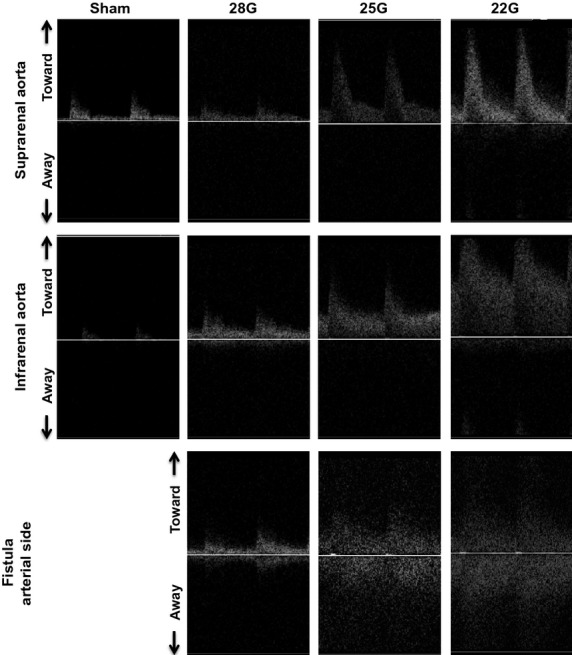
Aortic waveforms after AVF. Representative waveforms observed using Doppler ultrasound in the suprarenal aorta (first row) and the infrarenal aorta (second row) proximal to the fistula, as well as in the aorta at the level of the fistula (third row). First column, sham procedure; second column, 28G fistula; third column, 25G fistula; fourth column, 22G fistula. The presence of a wide range of frequencies at each time point is characteristic of disturbed flow.

**Figure 7:**
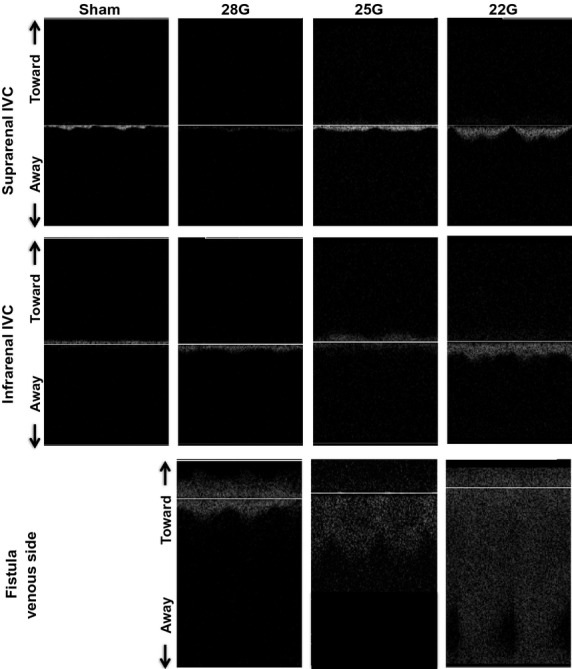
IVC waveforms after AVF. Representative waveforms observed using Doppler ultrasound in the suprarenal IVC (first row) and the infrarenal IVC (second row) distal to the fistula, as well as in the IVC at the level of the fistula (third row). First column, sham procedure; second column, 28G fistula; third column, 25G fistula; fourth column, 22G fistula. The presence of a wide range of frequencies at each time point is characteristic of disturbed flow.

Figures6 and 7 show representative waveforms in the aorta proximal to the fistula and the IVC distal to the fistula, respectively. In the aorta proximal to the fistula, the magnitude of the pulsatile SS, both PSV and EDV increased with increased diameter of the fistula (Fig.6; upper row, suprarenal aorta; middle row, infrarenal aorta); waveforms at the fistula (Fig.6; bottom row) show further increased magnitude of SS velocity. All these waveforms demonstrate both forward and reversed flow, that is, increased range of SS frequencies, consistent with the presence of disturbed flow in the aorta proximal to the fistula and at the fistula. Flow patterns in the IVC distal to the fistula were similar to those seen in the aorta proximal to the fistula although of much less magnitude; similarly, the flow at the fistula was consistent with disturbed SS, with increased magnitudes and frequencies of SS proportional to the increased fistula diameter (Fig.7).

### Klf2 expression in the aorta proximal to the fistula

**Figure 8:**
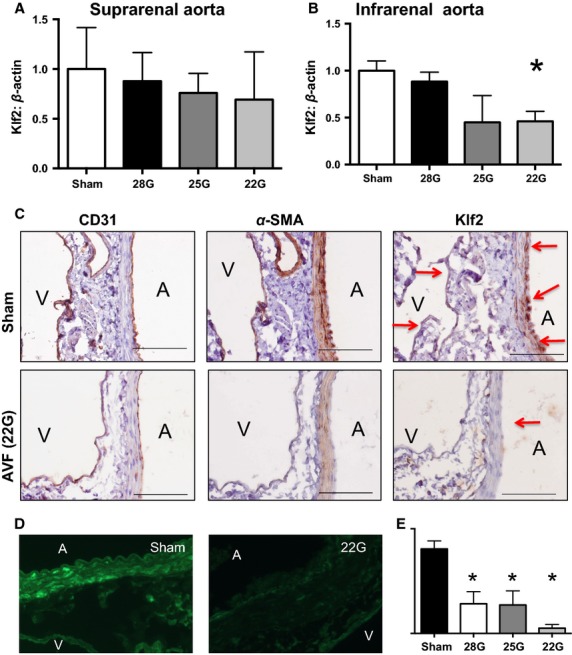
Klf2 expression in the aorta proximal to the fistula. (A,B) Densitometry (top) and representative images (bottom) of Klf2 protein expression in Western blots derived from whole vessel lysates of the suprarenal (A) or infrarenal (B) aorta. Klf2 density was normalized to that of beta actin. *n* = 5 for each group. A, *P* = 0.57, ANOVA; B, *P* < 0.0001, ANOVA. *P < 0.05 compared to the sham group, post hoc test. Primary antibody used was ab189541 (Abcam). C) Representative photomicrographs show immunohistochemistry analysis of AVF in mice after sham or AVF surgery; examining specimens for CD31 (first column), alpha-actin (second column), or Klf2 (third column). Scale bar, 100 *μ*m. Primary antibody used was sc-18690 (Santa Cruz Biotechnology). Red arrows points out the positive staining for Klf2. D) Representative images of immunofluorescence examination for Klf2 in the infrarenal aorta proximal to the fistula. Primary antibody used was LS-B5627 (LifeSpan Biosciences, Inc). E) Quantification of Klf2 intensity of figure D. *P* < 0.0001, ANOVA; *P < 0.05 compared to the sham group, post hoc test. *n* = 2 for each group, measured at three points per sample.

Since Klf2 expression is increased with laminar SS but may be decreased with disturbed SS, we determined the expression of Klf2 in the aorta proximal to the fistula where we directly observed increased magnitude of disturbed SS (Figs.6) (Wang et al. 2006; Zhang and Friedman 2012; Davies et al. 2013). There was a trend toward decreased Klf2 expression in larger diameter fistulae in the suprarenal aorta (Fig.8A) and a significant decrease in the infrarenal aorta (Fig.8B). Immunohistochemistry analysis of the aorta proximal to the fistula showed that Klf2 is present in the aortic endothelium of the sham group (Fig.8C, upper rows) and downregulated in the endothelium proximal to the AVF produced with a 22-gauge needle (Fig.8C, lower rows). Similar results were obtained with AVF produced with either 28-gauge or 25-gauge needles (data not shown). Since some Klf2 immunoreactivity was present in the media as well as the endothelium, we verified the reduced intimal staining using a different antibody to Klf2; similar results were obtained (data not shown). Since both western blot and immunohistochemistry analyses suggest diminished Klf2 protein expression in the aorta proximal to the fistula, we quantified Klf2 protein expression using immunofluorescence (Fig.8D). Immunofluorescence confirmed reduced Klf2 immunoreactivity in the aortic wall proximal to the fistula, with diminished intensity proportional to fistula diameter (Fig.8E).

**Figure 9:**
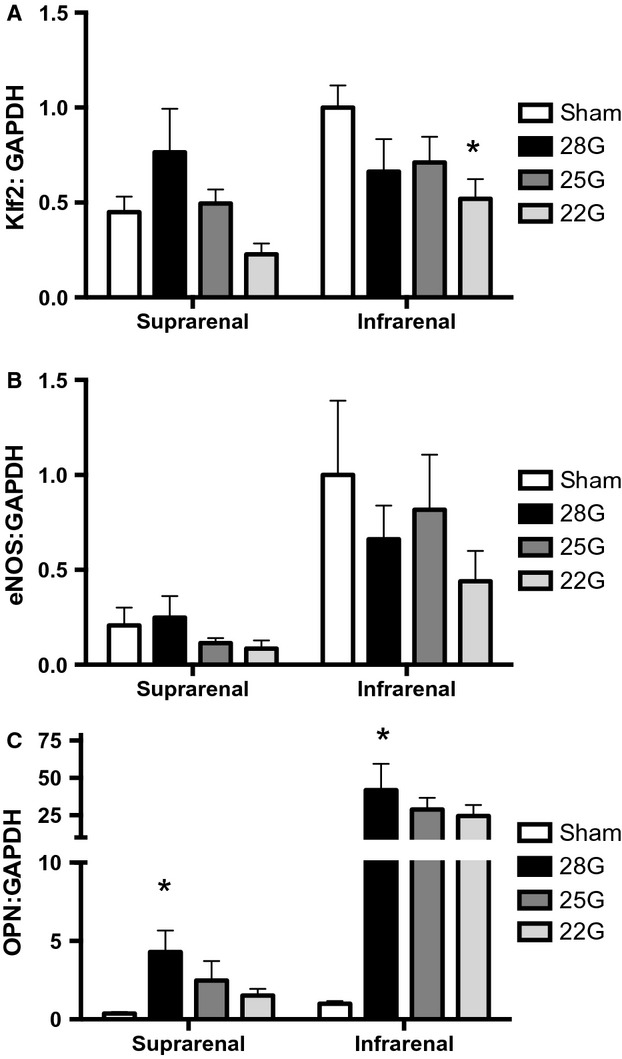
mRNA expression in the aorta proximal to the fistula. Quantification of Klf2 (A), eNOS (B) and osteopontin (OPN) (C) mRNA expression in the suprarenal and infrarenal aorta proximal to the fistula. All genes were normalized to GAPDH expression. *N* = 5–10 A) *P* = 0.03 (2-way ANOVA); *P = 0.02 post hoc test. B) *P* = 0.0003 (2-way ANOVA); C) *P* = 0.005 (2-way ANOVA); *P = 0.005 post hoc test.

Since Klf2 protein expression is reduced in the aorta proximal to the fistula (Fig.8), we determined the pattern of Klf2 mRNA expression in the aorta proximal to the fistula. Klf2 mRNA expression decreased in the aorta proportionally to fistula diameter (Fig.9A), a similar trend as seen with Klf2 protein expression. We also examined the mRNA expression of eNOS that is downstream from Klf2; eNOS expression also showed the same trend in the aorta proximal to the AVF (Fig.9B). To determine whether mRNA expression was globally decreased after AVF creation, we examined the expression of osteopontin that increases its expression in patent AVF (Abeles et al. 2006); osteopontin expression increased more than two fold in the suprarenal aorta and over 20 fold in the infrarenal aorta (Fig.9C), consistent with selective diminished expression of Klf2. These results are consistent with diminished mRNA expression of Klf2 in vivo in response to disturbed SS.

## Discussion

We report that modification of the standard 25-gauge mouse aortocaval fistula model of AVF with a larger (22-gauge) needle produces larger diameter fistulae (Figs.3) with increased SS magnitudes and range of frequencies (Figs.6) consistent with disturbed SS in the aorta proximal to the fistula on postoperative day 1. Klf2 protein (Fig.8) and mRNA (Fig.9) expression decreases in the infrarenal aorta proximal to the fistula, consistent with the presence of disturbed SS in vivo. These results are consistent with the presence of disturbed SS in the AVF immediately after surgery, and that Klf2 expression is diminished in response to disturbed SS in vivo.

Successful hemodialysis requires a functional access. AVF are the preferred conduit, but must mature postoperatively, for example, dilate, thicken and increase flow, to allow successful function (Abbruzzese et al. 1998; Allon and Robbin 2002; Rothuizen et al. 2013). Although many factors have been proposed as predictors of AVF maturation (Lauvao et al. 2009; Voormolen et al. 2009; Smith et al. 2012), the impact of a potentially optimal diameter of the anastomosis is still controversial due to the difficulties of surgical standardization, postoperative surveillance, as well as variations in human anatomy and physiology. It is intuitive that a larger diameter anastomosis could give better AVF patency but also may lead to steal syndrome or even cardiac failure. Our finding that disturbed frequencies of SS are present at the AVF in vivo suggests another potential etiology for failure of AVF maturation as well as a factor that could potentially be optimized.

We altered SS in vivo by modification of our previously reported AVF model to use different needle diameters (Yamamoto et al. 2013a,b). This method of creating an AVF is simple, reproducible and alters SS in vivo, similar to other models (Li et al. 2007; Nam et al. 2009; Shin et al. 2013). As expected, this model showed increased SS in the arterial limb proximal to the AVF (Fig.4E and F) but not in the venous limb distal to the AVF (Fig.5E and F). These findings are similar to a report in human AVF, and reflect the complexity of hemodynamic forces after surgery (McGah et al. 2013). In addition, a computational fluid dynamics simulation showed that disturbed SS, with areas of oscillatory flow, localized to the point of AVF stenosis (Ene-Iordache and Remuzzi 2012). We show that disturbed flow is present at the fistula (Figs.7, bottom rows), which is the point at which stenosis forms by postoperative day 42 in this model (Yamamoto et al. 2013b). In addition, this model provides an opportunity to examine the expression of Klf2 in vivo, and specifically to determine whether increased SS magnitude or range of frequencies is the predominant influence on Klf2 expression. Klf2 expression increases with exposure to laminar SS (Wang et al. 2006; Zhang and Friedman 2012; Davies et al. 2013); however, Klf2 expression decreases with exposure to disturbed SS (Wang et al. 2006; Chien 2007; Ni et al. 2010; Lee et al. 2012). Our results are consistent with these reports, showing decreased mRNA (Fig.9) and protein (Fig.8) expression in this model, suggesting that the disturbed character of the SS frequencies has greater effect on Klf2 expression than the increased magnitudes of SS. Interestingly, Klf2 and eNOS expression differed in the suprarenal and infrarenal aorta (Fig.9); these differences are likely due to the different shear stress frequencies between these two areas of the aorta, as they are separated by the celiac, superior mesenteric and renal arteries that each create flow disturbance as the blood travels distally.

Although Klf2 is specific to the endothelium (Wang et al. 2006), our immunohistochemistry and immunofluorescence studies, using two different antibodies, show some minor immunoreactivity in the media. Although the significance of this finding is not clear, it is possible that the mouse media have some cross-reactivity with the antibodies; nevertheless, the downregulation of Klf2 protein with disturbed SS is consistent among all these studies as well as with the downregulation of Klf2 mRNA.

## Conclusion

We show that using a larger diameter needle creates a larger diameter anastomosis in the mouse AVF model. Increased SS magnitude and range of frequencies creates disturbed SS in vivo that is associated with diminished Klf2 mRNA and protein expression. These results suggest that Klf2 expression is predominantly regulated by SS frequency, rather than magnitude, and additionally suggest potential new targets to improve AVF maturation.

## Conflict of Interest

None declared.
